# Statement of Retraction: Regulation function of MMP-1 downregulated by siRNA on migration of heat-denatured dermal fibroblasts

**DOI:** 10.1080/21655979.2020.1770001

**Published:** 2020-05-21

We, the Editor, Publisher and authors have retracted the following article from *Bioengineered*:

Xianghui He, Jinhua Dai, Youfen Fan, Chun Zhang & Xihong Zhao, Regulation function of MMP-1 downregulated by siRNA on migration of heat-denatured dermal fibroblasts, *Bioengineered* (2017) 8(6): 333-346, DOI:10.1080/21655979.2016.1267885

The above article has been retracted at the request of the authors who have, subsequent to publication, found that the experiments reported are not reproducible and that the conclusions made are therefore unreliable.

We have been informed in our decision-making by our policy on publishing ethics and integrity and the COPE guidelines on retractions.

The retracted article will remain online to maintain the scholarly record, but it will be digitally watermarked on each page as ‘Retracted’.

